# Should the dose of levothyroxine be changed in hypothyroidism patients fasting during Ramadan?

**DOI:** 10.3906/sag-1911-28

**Published:** 2020-06-23

**Authors:** Arzu OR KOCA, Murat DAĞDEVİREN, Mustafa ALTAY

**Affiliations:** 1 Department of Endocrinology and Metabolism, University of Health Sciences,Keçiören Health Administration and Research Center, Ankara Turkey

**Keywords:** Fasting, hypothyroidism, levothyroxine therapy, Ramadan

## Abstract

**Background/aim:**

Muslims worship by fasting from predawn (suhoor) until sunset (iftar) for 30 days in the religious month of Ramadan. In addition to prolonged hunger, patients fasting with a diagnosis of hypothyroidism take their doses of levothyroxine (LT4) outside of daytime fasting hours. The purpose of our study is to compare the values of hypothyroid patients which have been obtained through thyroid function tests before and after Ramadan.

**Materials and methods:**

Ninety-seven patients; ranging from 18 to 65 years old, who were followed with a diagnosis of hypothyroidism, who fasted during Ramadan, and who had no change of their LT4 dose for at least 6 months were included in the study.

**Results:**

The median serum thyroid-stimulating hormone (TSH) level of patients prior to fasting was 2.19 mIU/L, while median serum TSH after fasting was 2.73 mIU/L. Serum TSH values after Ramadan increased significantly compared to those prior to Ramadan (P = 0.004).

**Conclusion:**

Our study demonstrates a significant increase in serum TSH levels after Ramadan but no significant change in serum free thyroxine (fT4) levels in hypothyroidism patients who are fasting. It may be appropriate to take precautions by making a small increase in LT4 dose before Ramadan in some hypothyroid patients wishing to fast.

## 1. Introduction

Hypothyroidism is a disease involving a metabolic deceleration as a result of insufficiency or rarely deficiency of thyroid hormone at the tissue level. Primary hypothyroidism is a widespread endocrinological disease appearing due to causes originating from insufficiency of the thyroid gland. The basis of treatment of hypothyroidism is the replacement by levothyroxine (LT4). It is recommended that it should be taken with some amount of water at least 30 min before breakfast on an empty stomach in order to prevent drug interaction and to increase intestinal absorption [1,2]. Because the absorption of LT4 is disturbed by some foods and oral drugs (cholestyramine resin, sucralfate, iron sulphate, calcium preparations, aluminium antacids, raloxifene, active charcoal, various soy products, and some herbal products), taking it on an empty stomach is important [3]. It may be taken on an empty stomach while going to sleep or 3–4 h after the last meal in some special situations [2]. While LT4 is absorbed at 80% when taken on an empty stomach, this rate decreases to 60% when taken on a full stomach [4].

Some Muslims throughout the world worship by fasting from predawn (suhoor) until sunset (iftar) for 30 days in the religious month of Ramadan. Patients may have difficulty in taking LT4 on an empty stomach during this fasting [5]. The month of Ramadan follows the Islamic calendar, which is 11–12 days shorter than the solar year, so the dates of Ramadan change every year and migrate through the seasons [6]. Fasting times of approximately 15–17 h occurred in Ramadan in 2018. Some small metabolic and hormonal changes may occur as a result of this prolonged fasting in Ramadan. Therefore, minimal changes are seen in the levels of serum thyroid-stimulating hormone (TSH), free triiodothyronine (fT3), and free thyroxin (fT4) in healthy individuals during Ramadan. However, it has been reported that patients with thyroid diseases could fast without needing any adjustment of treatment and without having any health dangers [7]. While several studies showed significant decreases in fT4 levels during Ramadan, it was demonstrated in some other studies that TSH increased in males, although TSH and fT4 levels remained within the normal ranges. It was reported that most of those changes were reversed again after Ramadan [8,9]. It was also shown that there is a positive relationship between fT4 level and the number of days fasting in females [7]. In a study conducted by Bolk et al., lower serum TSH levels were determined in patients taking LT4 before going to bed [10].

Interest in the medical aspects of fasting has increased in the last 30 years [11,12]. In particular, studies on the management of patients with diabetes mellitus have been focused, but the studies focusing on the medical management of thyroid diseases in relation to Ramadan fasting are limited [7,13].

For this reason, we aimed to observe changes in thyroid function tests before and after the month of Ramadan in hypothyroidism patients who were fasting.

## 2. Materials and methods

A total of 97 patients between the ages of 18 and 65 years old were included in the study. They had all applied to the Clinic of Endocrinology and Metabolic Diseases, Keçiören Education and Research Hospital of the University of Health Sciences, in 2018, and were followed with a diagnosis of hypothyroidism and were fasting for Ramadan in 2018. Eighty-one patients had Hashimoto hypothyroidism, 14 had iatrogenic hypothyroidism (6 had postoperative papillary thyroid cancer, 8 had postoperative benign multinodular goitre) and 2 had postradioactive iodine ablation hypothyroidism. TSH levels were determined individually considering the etiologic causes, age, and comorbidities of the patients. Patients whose TSH levels were at the determined target and, therefore, who did not undergo LT4 dose change for the last 6 months were included in the study. Patients regularly fasting for at least 20 days were included in the study, and thyroid functions (TSH, fT4) of patients within the last 1 month before Ramadan and within the month after Ramadan were recorded. After informed consent was obtained from the patients meeting the inclusion criteria and attending routine check-ups in the outpatient clinic, age, sex, daily and weekly intake dose of LT4 treatment, fasting patterns if they were fasting in the month of Ramadan, and time of drug intake were recorded. Based on their biochemical tests or examinations; patients with known malignant disease other than thyroid papillary cancer, those fasting while pregnant, those fasting irregularly during Ramadan and the patients who vary their daily time of LT4 consumption, and those using another drug together with LT4, those receiving proton pump inhibitory therapy, those using a known drug that effects intestinal absorption of LT4, those suspicious with subacute thyroiditis and with known liver or kidney failure were excluded from the study. Patients with overt hypothyroidism (TSH ≥10 mIU/L) or overt thyrotoxicosis (TSH ≤ 0.1 mIU/L) prior to Ramadan were not included in the study despite receiving replacement therapy due to hypothyroidism. 

Serum TSH and fT4 measurements were performed using chemiluminescence on an automatic Abbott i2000 immunoassay device. The TSH reference interval is 0.27–4.3 mIU/L; the fT4 reference interval is 0.75–2.15 ng/dL.

### 2.1. Statistical analysis

Mean, standard deviation, median, minimum, and maximum values have been given in definitive statistics related to continuous data and percentage values have been given for categorical variable. The Shapiro–Wilk test has been utilized for examining the suitability of data to normal distribution. The Mann–Whitney U test has been used for comparing the time of drug consumption (suhoor or iftar), and the Wilcoxon test has been used for comparing measured TSH and fT4 values before Ramadan and after Ramadan. The Spearman correlation coefficient has been utilized for examining the association between daily LT4 dose and TSH. The IBM SPSS Statistics 20 (IBM Corp, Armonk, NY, USA) program has been used in evaluations, and P < 0.05 has been accepted as the limit for statistical significance.

## 3. Results

The mean age of the 97 patients included in the study was 45 (18–65) years; 92.8% of patients were female and 7.2% were male. The mean LT4 dose of patients was 82.14 (12.5–175) µg/day. It was determined that 10.3% of patients took their drugs at iftar time and 89.7% at suhoor (Table). 

**Table:** Demographic characteristics and laboratory parameters of participants

	Values,measurement results
Sex- n (%)	
Female	90 (92.8)
Male	7 (7.2)
Age - years	45 (18–65)
Levothyroxine dose (µg/day)	82.14 (12.5–175)
Time of drug consumption - n (%)	
Iftar	10 (10.3)
Suhoor	87 (89.7)
Serum TSH (mIU/L)	
Before fasting	2.19 (0.14–9.09)
After fasting	2.73(0.01–21.21)
fT4 (ng/dL)	
Before fasting	1.11 (0.76–1.41)
After fasting	1.11 (0.78–2.14)

Abbreviations: TSH: thyroid stimulating hormone, fT4: free thyroxin.

The median serum TSH of patients before fasting was 2.19mIU/L (0.14–9.09), and median serum TSH after fasting was 2.73mIU/L (0.01–21.21). Serum TSH levels after the month of Ramadan increased significantly compared to the levels before Ramadan (P = 0.004). The median fT4 of patients before fasting was 1.11ng/dL (0.76–1.41), and median fT4 after fasting was 1.11ng/dL (0.78–2.14). No difference was found between fT4 values before and after the month of Ramadan (P > 0.05) (Figure).

**Figure:**
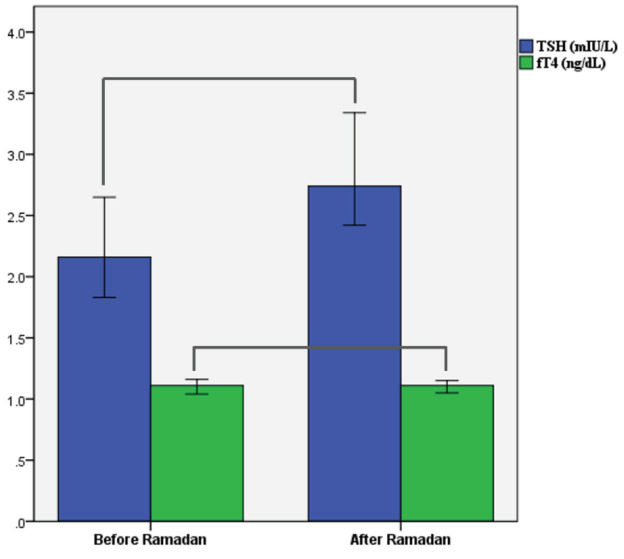
Graph of difference for thyroid functions before and after the month of Ramadan.Abbreviations: TSH: thyroid-stimulating hormone, fT4: free thyroxin.

The median serum TSH of patients taking LT4 at iftar time was 1.35mIU/L (0.20–4.48) before the month of Ramadan and was 2.98mIU/L (0.79–12.84) after Ramadan. The median TSH of patients taking LT4 at suhoor was 2.24 mIU/L (0.14–9.09) before the month of Ramadan and 2.73mIU/L (0.01–21.21) after Ramadan. There was a statistically significant increase in serum TSH levels in both patients taking the drug at iftar and those taking it at suhoor (P = 0.01 and P = 0.03, respectively). The median value of serum TSH change from before Ramadan to after Ramadan was 1.87mIU/L (–0.49 to 12.04) in those taking LT4 at iftar, while it was 0.36mIU/L (–7 to 14.53) in those taking LT4 at suhoor. No difference was found between increase of serum TSH in those taking LT4 at iftar and those taking it at suhoor (P > 0.05).

The median fT4 of patients taking the drug at iftar time was 1.16 ng/dL (0.85–1.38) before the month of Ramadan, while the median serum fT4 of those taking it at iftar time was 1.07 ng/dL (0.88–2.14) after the month of Ramadan. The fT4 median of patients taking the drug at suhoor time was 1.10 ng/dL (0.76–1.41) before Ramadan, while it was 1.10 ng/dL (0.78–1.72) after Ramadan. No significant change was determined between fT4 values before and after the month of Ramadan in patients taking LT4 at either iftar or suhoor (P > 0.05).

From the correlation analysis, a negative correlation was found between the daily drug dose consumed and serum TSH values after the month of Ramadan (r = –0.257, P = 0.01). A positive correlation was found between daily drug dose and fT4 values after the month of Ramadan (r = 0.438, P < 0.001).

## 4. Discussion

This study is the first study examining the influence of Ramadan fasting on thyroid function tests in hypothyroidism patients who are fasting in Turkey. In our study, we found that the median TSH level increased significantly at the end of Ramadan, whereas fT4 did not change.

Changes in gastric motility due to prolonged fasting and the relieving of hunger with heavy meals may cause fluctuations of the circadian rhythm. Therefore, deiodinase activity and consequently LT4 metabolism may also be influenced [14,15]. Raza et al. argued that LT4 intake at bedtime prevents those problems and is effective in reducing TSH levels for this reason [7]. Among our patients, 10.3% were taking their drugs at iftar time and 89.7% at suhoor time, and it was seen that there was no difference between drug intake at iftar or suhoor in terms of increases in TSH values. No patient was taking their drugs immediately before sleeping in our study. The increase in serum TSH values after the month of Ramadan occurring in both patients taking LT4 at iftar and those taking it at suhoor suggests that this increase is caused by drug intake without letting the stomach empty after prolonged hunger and disturbance of drug absorption.

Karoli et al., in their study including 49 patients, stated that the drug should be taken by waiting at least 2 h between a meal and the consumption of the drug. However, it was determined in the same study that approximately 75% of the patients fasting in Ramadan did not wait long enough between the meal and drug intake [5]. In our study, it is thought that the increase in TSH values of fasting patients may have been caused by their eating iftar or suhoor meals and then not waiting long enough before drug intake.

The limited data obtained from the small number of studies published regarding hypothyroidism and Ramadan fasting recommend to increase the LT4 dose by 25–50 µg/day at the beginning of the month of Ramadan and to continue the increased dose for up to 15–20 days after Ramadan [16]. This approach is thought to prevent fluctuations in the TSH values of fasting patients. When our study results are taken into consideration, we think that this approach is also suitable for our patient population. 

Some of the published literature has indicated that LT4 intake 1 h before suhoor is suitable in the month of Ramadan, but most patients had difficulty in waking up that early and/or missed the dose and they took their drugs together with their meals. Drug intake on a full stomach has been found to decrease absorption by 20% [1]. However, there are also data suggesting that LT4 intake in the morning or in the evening had no influence on serum TSH and quality of life [17].

Sheikh et al. showed that the mean TSH level in fasting patients with hypothyroidism increased by 2.32 ± 3.80 mIU/L after the month of Ramadan. While the median increase of that parameter in our study was 1.87 mIU/L in patients taking LT4 at iftar, it was 0.36 mIU/L in those taking LT4 at suhoor. However, Sheikh et al. argued differently than our thought that the increase in TSH had not been influenced by the timing of LT4 intake and meal intervals [6]. 

Although hypothyroidism is a common disease, there are a limited number of studies evaluating thyroid functions of patients with the diagnosis of hypothyroidism who are fasting during Ramadan. When those studies are examined in terms of the number of patients, it is seen that they were conducted with small numbers of individuals [5,6,9,18]. We think that our study is more powerful in terms of the ability to generalize because 97 patients were evaluated in our study. By indicating that thyroid functions of patients evaluated in this study were at target values in the last 6 months by using a stable dose of LT4, the absence of a requirement for a change of LT4 dose points out the importance of the increase in serum TSH levels that we determined after the month of Ramadan.

Although our study was conducted in a large education and research hospital, it is a single-centre study and may not represent the general population. For this reason, the generalizability of the results may be limited. The medical histories of patients were obtained during control visits at the outpatient clinic within 1 month after Ramadan and the correctness of compliance by verbal declaration may have low reliability. That is another limitation of our study. The number of male individuals and the number of individuals taking their drugs at iftar were low in our study. In addition, our patients who had menstruation in their reproductive age were also in our study population. The change in oestrogen and progesterone levels due to prolonged hunger and the effect of the affected menstruation on TSH could not be evaluated so, this is an another limiting factor for our study.

 In conclusion, our study demonstrates that there is not a significant change in fT4 levels of hypothyroidism patients after the month of Ramadan, while an increase occurred in TSH levels. This increase may be of particular importance in special patient populations such as pregnancy or papillary thyroid cancer, where TSH target values are in the narrower range, and in groups of patients where TSH levels are closer to the high target values. Therefore, it may be appropriate to take precaution by making a small increase in LT4 dose before Ramadan in some hypothyroid patients wishing to fasting. However, further research is needed to fully understand in which patients and in what amount the LT4 dose increase will be appropriate and at what time it is appropriate to take this dose.

## Acknowledgements

Approval has been obtained from the Ethics Committee of Clinical Research at Keçiören Education and Research Hospital, University of Health Sciences (project no: KAEK-15/1708). All procedures performed in studies involving human participants were in accordance with the ethical standards of the institutional and/or national research committee and in line with the Helsinki Declaration (1964) and its later amendments or comparable ethical standards. Informed consent was obtained from all individual participants included in the study and no funding is received for this study. All authors contributed at every stage of the study.
